# Time-resolved X-ray Tracking of Expansion and Compression Dynamics in Supersaturating Ion-Networks

**DOI:** 10.1038/srep17647

**Published:** 2015-12-14

**Authors:** Y. Matsushita, H. Sekiguchi, K. Ichiyanagi, N. Ohta, K. Ikezaki, Y. Goto, Y. C. Sasaki

**Affiliations:** 1 Graduate School of Frontier Sciences, The University of Tokyo, 5-1-5 Kashiwanoha, Kashiwa City, Chiba, Japan; 2 Japan Synchrotron Radiation Research Institute (JASRI) 1-1-1, Kouto, Sayo-cho, Sayo-gun, Hyogo 679-5198 Japan; 3 High Energy Accelerator Research Organization, Photon Factory, 1-1 Oho, Tsukuba City, Ibaraki, Japan; 4 Institute for Protein Research, Osaka University,3-2, Yamadaoka, Suita City, Osaka, Japan

## Abstract

Supersaturation of a solution system is a metastable state containing more solute than can be normally solubilized. Moreover, this condition is thermodynamically important for a system undergoing a phase transition. This state plays critical roles in deposition morphology in inorganic, organic, polymer and protein solution systems. In particular, microscopic solution states under supersaturated conditions have recently received much attention. In this report, we observed the dynamic motion of individual ion-network domains (INDs) in a supersaturated sodium acetate trihydrate solution (6.4 M) by using microsecond time-resolved and high accuracy (picometre scale) X-ray observations (diffracted X-ray tracking; DXT). We found that there are femto-Newton (fN) anisotropic force fields in INDs that correspond to an Angstrom-scale relaxation process (continuous expansion and compression) of the INDs at 25 μs time scale. The observed anisotropic force-field (femto-Newton) from DXT can lead to new explanations of how material crystallization is triggered. This discovery could also influence the interpretation of supercooling, bio-polymer and protein aggregation processes, and supersaturated systems of many other materials.

Supersaturation is widely known to play a critical role in the process of crystal nucleation and growth from solution. This condition is thermodynamically important for solution systems undergoing phase transitions. Nucleation processes in supersaturated solutions have been characterized intensively in various scientific fields, including crystal morphology control^1^, elucidation of the complex bio-mineralization process of calcium carbonate^2^,^3^,^4^, improved performance of thermal storage systems using phase change materials^5^ and abnormal protein aggregation, which is known as the causative agent of some neurological disorders^6^,^7^.

Recently, the characterization of prenucleation clusters (PNCs) in supersaturated solutions has received much attention^3^. PNCs are considered to be molecular precursors to the nucleation phase in solution. PNC sizing has been investigated by experimental studies with ultracentrifugation^2^, Raman spectroscopy^8^, cryo-transmission electron microscopy, scanning electron microscopy^1^,^3^,^9^ and molecular dynamics simulations^2^. PNCs as solute-assembled structures have been reported in various supersaturated solutions, such as calcium carbonate, urea, citric acid, and K_2_SO_4_ with critical sizes of approximately 1–10 nm^8^. These small cluster properties are considered to be important for controlling crystal morphology. The mechanism behind urea crystal formation has been reported by Parinello *et al.* using MD simulations. They have demonstrated the growth mechanism of two different crystal faces ({001} and {110}) of the urea crystal and the effect of additives on crystal growth. These studies have revealed that crystal morphology can be controlled by inhibition of crystal face growth by additives during stable nucleus cluster production in supersaturated solutions^10^. Protein crystallization mechanisms may also be explained by a two-step process. Vekilov *et al*. have observed dense liquid clusters in lysozyme and glucose isomerase solutions at precursor stages of crystal nucleation by applying dynamic light scattering. These cluster sizes vary from several tens to several hundreds of nanometres^11^,^12^. However, experimental studies of the dynamic properties of PNCs have not been reported as extensively because PNCs are presumably highly dynamic entities and their molecular rearrangement processes are extremely fast.

The molecular relaxation of a polymer/water solution at the glass transition state has been intensively studied, and it has been shown that various molecular rearrangement dynamics coexist in such solution systems^13^,^14^.

In this study, we developed a novel method for dynamic observation of local supersaturated solutions at microsecond time scales using an X-ray single molecule methodology, also known as diffracted X-ray tracking (DXT)^15^,^16^,^17^. In particular, DXT can be used to detect high accuracy rotational angular observations in solution systems. DXT is a promising means to acquire novel dynamic information from supersaturated solutions.

We utilized DXT as a high time resolution (μs) and high positional accuracy (pm)^17^ X-ray single molecule observation method to investigate the properties of supersaturated solutions (Fig. 1a). The assembled solute clusters of supersaturated solutions are drawn as ion-network domains (INDs). DXT measures the rotational angular displacement of dissolved gold nanocrystals by tracking X-ray diffraction spots (Fig. 1b). Additionally, each trajectory can be converted into two-axis (θ and χ) directional angular displacements. Because it is very easy to maintain metastable supersaturated solution states at room temperature even if the solution contains dispersed gold nanocrystals, we chose saturated (6.0 M) and supersaturated (6.4 M) sodium acetate trihydrate^18^. Trajectories of Laue diffraction spots from gold nanocrystals in saturated and supersaturated solutions were measured at 25 μs time scale resolution. The trajectories tracked from saturated and supersaturated conditions totalled 184 and 267 diffraction traces, respectively (Fig. 1c).

The time-resolved (25 μs) rotational angular displacements in the θ and χ directions over 1,000 μs are shown in Fig. 1d,e. From this result, χ directional dynamics reached approximately 100 mrad as a maximum angular displacement (Fig. 1d,e). However, the maximum θ directional angular displacement was only 20 mrad under both conditions. (Fig. 1d,e). From these data, we selected χ directional dynamics analysis to identify the difference between supersaturated and saturated conditions.

For a detailed analysis of gold nanocrystal dynamics, we conducted statistical processing by using two-dimensional histograms of the θ and χ directions for 25, 150 and 300 μs (Fig. 2a,b). To calculate the characteristic dynamics of gold nanocrystals under supersaturated conditions, we performed a subtraction process for two-dimensional histograms by subtracting the supersaturated condition from the saturated one (Fig. 2c). From this result, we confirmed that the subtracted two-dimensional histogram was separated into two different motional groups at 300 μs, and these groups were especially changed in the χ direction.

In a more precise analysis, we sought to determine χ directional dynamics and attempted log-normal distribution regression analysis^19^ in the distribution of saturated and supersaturated conditions in log scale angular displacement (Fig. 2d,e, Supplementary Fig. S1a,b, Supplementary Table S1). Whereas the distribution of the saturated condition (Fig. 2d) followed a log-normal distribution in each time axis, the supersaturated condition (Fig. 2e) showed two different peaks at 300 μs as confirmed by the subtracted two-dimensional histogram (Fig. 2c).

To analyse the time-resolved dynamical properties of the two different motional groups, we conducted an analysis based on mean square displacement (MSD)^20^ for each peak position. The square of each separated peak position dependent on time was plotted as shown in Fig. 2f. These results confirmed that the MSD form of each peak position could be classified into different diffusion modes. The MSD curve of the high displacement peak position (H) increased parabolically. In contrast, the MSD curve of the low displacement peak position (L) reached a limit of 3.50 mrad^2^ at 100 μs. From this result, we distinguished two different diffusion modes in supersaturated solutions. In particular, the coexistence of different dynamics are similar to the phenomena for the water molecular relaxation process in a polymer/water solution at the glass transition point^13^. The integrated area ratios of the low and high displacement peaks were approximately 0.9 and 0.1, respectively. The coexistence of these different dynamic modes explains a physical dynamics model of INDs in the vicinity of an individual single gold nanocrystal (Fig. 2g). From the curve forms of L and H from MSD analysis, L indicates the restricted appropriate rotational dynamics of gold nanocrystals from isotropic force pressure (Fig. 2g) in expansion and contraction dynamics of INDs located in the vicinity of gold nanocrystals. However, the curve form of H increases parabolically and indicates extremely small anisotropic force pressure generation (Fig. 2g). This curve is fit by the equation of direct motion with diffusion: *MSD* (*t*)* = 4Dt + *(*Vt*)^2^, where *MSD* corresponds to the square of angular displacement (mrad) of the gold nanocrystal^20^, *D* is the rotational diffusion constant, *V* is the rotational velocity and *t* is elapsed time. The values of *D* and *V* from regression analysis by this equation were obtained as 0.28 ± 0.03 (mrad^2^/μs) and 0.064 ± 0.003 (mrad/μs), respectively (Supplementary Table S3a). From the obtained value of *V*, we calculated the gold nanocrystal anisotropic force pressure value affected by non-uniform expansion and contraction dynamics of INDs (Fig. 2g), where the gold nanocrystal size was estimated as approximately 100 nm. To calculate the anisotropic observed force pressure *P*_*ob*,_ we used the equation *P*_*ob*_ = Γ/h = *2πC*_*rd*_*W*/*h*. Here, Γ is the rotational torque (N m), *C*_*rd*_ = πηh^3^ is the rotational frictional drag coefficient, the viscosity coefficient of sodium acetate (6.4 M) *η* (3.18 × 10^−3^ kg/m s) was estimated from prior studies of sodium acetate solutions using Jones Dole equation^21^, *h* is the radius of a gold nanocrystal (50 nm), and *W* = *V*/2*π*.^22^. The values of obtained parameters are shown in Supplementary Table S3b. In addition, the observed anisotropic force pressure *P*_*ob*_ from DXT was 1.60 femto-Newtons (fN). This value was calculated on the basis of the assumption that the rotational centre is the centre of a gold nanocrystal (Supplementary Fig. S5). This extremely small pressure force could be a novel parameter for controlling crystallization morphology in supersaturated solution systems.

According to the Einstein and Navier-Stokes equations, there is a strong relationship between the size of labelled particles and their dynamics. The DXT utilizes labelling methodology, making it necessary to evaluate label size effects. The sizes of single gold nanocrystals were assigned from the observed intensities of Laue diffraction spots, and the intensities were normalized to the background scatter intensity (Supplementary Fig. S4). The χ directional angular displacement of gold nanocrystals during 25 μs time intervals depended on the normalized intensity in saturated and supersaturated solutions, as shown in Fig. 3c,d, respectively. The inset histograms show a recording of the section of χ directional angular displacement at 25 μs under a specified normalized intensity range. All histograms in each section are fitted to a log-normal distribution, and each peak position is an average from the fitting process. The peak positions are plotted as black circles. From a log-linear fitting process for the black circle plots, the intercept values (0 of Laue diffraction intensity) of the saturated and supersaturated conditions were 87.1 and 27.5 mrad per 25 μs, respectively. These values are considered to be the estimated original velocities of the sodium acetate solution for non-labelled DXT. In addition, we observed that gold nanocrystals in saturated solutions followed basic diffusion particle theory (fig. 3c). In contrast, the behaviour of gold nanocrystals in supersaturated solutions was largely independent of their size. These results are consistent because most gold nanocrystals are packed with INDs in high-density conditions.

To identify the existence of INDs, we performed small angle X-ray scattering (SAXS). SAXS data provide structural information about the average size and shape of large structures present in a system. The normalized scattering intensity I(q), which is dependent on the scattering vector q and is concentration dependent, was assessed in samples of three different concentrations: supersaturated (6.4 M), saturated (6.0 M) and unsaturated (3.0 M) solutions. Fitted parameters were obtained for each sample condition (Fig. 4b), where *n* is the slope, and A is the intercept value of log (*I(q)*) = *n q* + *A*. As shown in Supplementary Table S2, the value of the slope *n* for the supersaturated condition (6.4 M) was drastically changed compared to that for the saturated and unsaturated conditions. This change indicates the existence of larger structures such as INDs, which we suggest as a model for supersaturated solutions from our DXT analysis.

In this study, we observed the local structure of supersaturated solutions by analysing the rotational dynamics of individual single gold nanocrystals dispersed in supersaturated sodium acetate solutions. Gold nanocrystals dispersed non-homogeneously displayed two different diffusion modes of slow (L) and fast (H) dynamics in the sub-microsecond region (Figs 2f and 3b), and we observed and determined the extremely small scale parameter as fn scale anisotropic force generation in a sodium acetate supersaturated solution (6.4 M). The positional accuracy of rotational angular displacement measurements using DXT, which reaches picometre scales, is advantageous for observing the nanoscale dynamic structure of supersaturated solutions, including the properties of INDs.

We determined that time scales of the dynamics of INDs from DXT were on the microsecond scale. However, the expected time scales of molecular rearrangement processes of PNCs in previous studies have been estimated as sub-picosecond time scales. This difference is caused by the different sizes of the PNCs and INDs. In future studies, we plan to develop more high-speed observations in a sub-picosecond range^23^ and to observe various sample systems, such as calcium carbonate, polymers, supersaturated protein solutions and PNCs using DXT. Moreover, the observed anisotropic force-field from the DXT method may lead to new explanations of material crystallization triggering. Until now, the dominant trigger-like physical factors affecting a material’s crystallization were unknown. Our dynamic anisotropic force-field provides the first hints of the cause of the crystallization triggering. A study examining the control of this anisotropic force-field in INDs should be undertaken. Additionally, our discovery in INDs may also influence the interpretation of supercooling, bio-polymer and protein aggregation processes, and supersaturated systems of complex molecules such as protein solutions^11^,^12^.

## Methods

### Diffracted X-ray tracking (DXT)

Supersaturated solutions of sodium acetate (6.4 M) were prepared with 0.67 g of sodium acetate trihydrate (Wako, Tokyo, Japan) dissolved in 0.5 ml water with dissolved gold nanocrystals. Dissolution of insoluble solute was conducted by heating to 80 °C and slow, natural cooling at room temperature (25 °C). Saturated solutions (6.0 M) were prepared by extracting the supernatant of a 0.5 g/0.5 ml sodium acetate solution without heating. The measurement solution volume was 40 μL in a sample holder with 150 μm of X-ray transmission thickness. Gold nanocrystals were fabricated by epitaxial growth on cleaved KCl {100} (7 mm × 7 mm area) under 10^−4^ Pa vacuum conditions. With AFM (atomic force microscopy), 1,000 particles of gold nanocrystals on 100 μm^2^ KCl substrates were observed [14]. Water-dissolved gold nanocrystals were prepared by detaching gold nanocrystals on 18 KCl substrates and dissolving in 3.0 ml water. It was confirmed that supersaturated solutions do not crystallize after measurement with X-ray radiation. The experiment was performed at SPring-8 BL40XU (Hyogo, Japan). The setup of DXT measurements is shown in Supplementary Fig. S2. X-ray with a total flux of 10^13^ photons/sec and energy widths ranging from 14.0–16.5 keV were used for DXT measurements. The X-ray beam size 40 μm (vertical) by 150 μm (horizontal). The diffraction spot from a gold nanocrystal can be detected when the lattice planes of the gold nanocrystal satisfy Bragg’s law (2*d* sin*θ* = *n λ*, where *d* is the inter-planar spacing, *θ* is the angle between the incident beam and the relevant crystal planes, *n* is an integer, and *λ* is the wavelength of the incident beam). The X-ray diffraction spots from each nanocrystal were transformed to visible light through an X-ray image intensifier (150 mm in diameter, V5445P, Hamamatsu Photonics, Japan), and time-resolved observation was performed using a CMOS camera (1024 pixel × 1024 pixel, 25 μs/frame, SA 1.1, Photoron, Japan) with 10 ms X-ray exposure time. DXT measurements were performed at room temperature (25 °C). The distance of the sample to the detector was approximately 100 mm.

Custom software written for IGOR Pro (Wavemetrics, Lake Oswego, OR) was used to analyse the diffraction spot tracks and trajectories. The time series of the angular position of the gold nanocrystals on samples in θ and χ directions were smoothed with a 3-point moving average filter to reduce high-frequency noise. The intensity of the diffraction spots from gold nanocrystals varies depending on the angular position in θ direction of diffraction spots and the corresponding flux of incident X-rays. Therefore, the intensity of the diffraction spot was normalized by considering the sample X-ray background. We examined the trajectories from the diffraction spots from Au (111) to analyse the relationship between the angular velocity and normalized intensity in Supplementary Fig. S3.

### Small Angle X-ray Scattering (SAXS)

Supersaturated (6.4 M) and saturated (6.0 M) solutions of sodium acetate were prepared in the same way as the samples prepared for DXT measurement without gold nanocrystals. Unsaturated solutions (3.0 M) were prepared with a two-fold serial dilution of the saturated solution (6.0 M).

SAXS measurements were performed using a bending-magnet beamline BL40B2 (SPring-8, Japan). The X-ray scattering profiles were recorded using a 2D detector (Pilatus 100K, Dectris Switzerland) with an area of 33.54 × 83.764 mm. X-ray energies ranged from 12.4 keV. The X-ray beam size was 200 μm × 200 μm at the detector position. The sample thickness throughout the X-ray was adjusted by approximately 3 mm by the two quartz windows sandwich holder. The distance of the sample to the detector was 3 m. Measurements were performed with 300 s of exposure time. The temperature was maintained at 25 °C during the experiment. The scattering intensities of all samples were obtained from the following equation: *I (q)* = (*I*_*sol*_ *(q, c)/T*_*sol*_) – (*I*_*water*_ *(q)/T*_*water*_), where *I (q)* is the scattering intensity, and *T* is the transmitted X-ray intensity obtained from the gas–flow type ionization chamber. The subscripts ‘sol’ and ‘water’ refer to sample (6.4, 6.0, 3.0 M) and water, respectively. The scattering vector magnitude *q* was calibrated from the lattice spacing of collagen (*q* = 2*πk*/63.26 nm^−1^, where *k* is the refractive index). The measurements of the sample and water scattering were repeated in three separate experimental sessions for sample 6.4 M and two separate experimental sessions for 6.0 M and 3.0 M. The inset SAXS profiles were the averaged values.

The experiment of gold nanocrystal dissolution in a supersaturated solution as shown in Supplementary Fig. S4 was performed by dissolving 20 g of sodium acetate trihydrate in 10 ml water. The sample on the left was conducted by heating to 80 °C and gradually cooling without mixing the sample. The sample on the right was prepared as in the sample on the left but with mixing.

## Additional Information

**How to cite this article**: Matsushita, Y. *et al.* Time-resolved X-ray Tracking of Expansion and Compression Dynamics in Supersaturating Ion-Networks. *Sci. Rep.* **5**, 17647; doi: 10.1038/srep17647 (2015).

## Supplementary Material

Supplementary Figure S1 - S5 and, Table S1 - S2

## Figures and Tables

**Figure 1:**
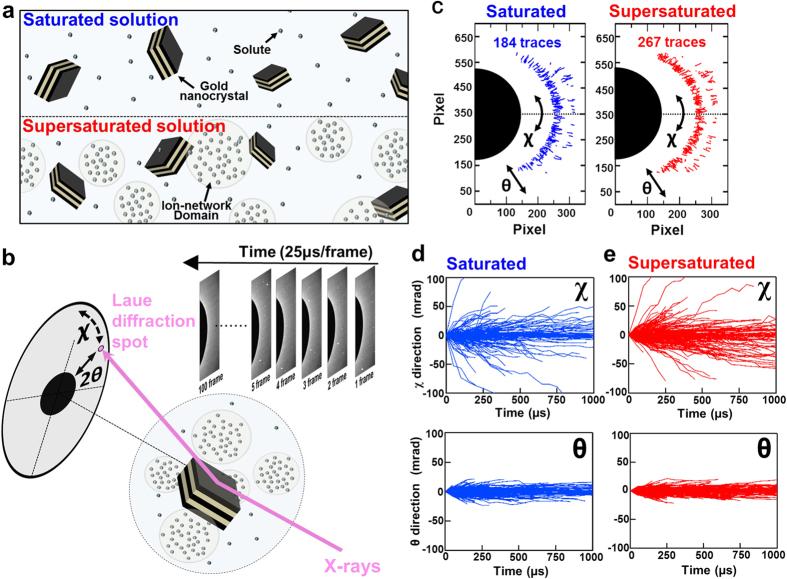
Schematic drawing of DXT measurements and obtained raw data from DXT. (**a**) Conceptual diagram of gold nanocrystals in saturated and supersaturated solutions. Gold nanocrystals are depicted with black and brown blocks stacked alternately. Dots indicate sodium acetate solutes, and the circles that surround dots express the INDs like a high-density liquid phase containing the number of solute. (**b**) Schematic drawing of Laue diffraction spots from a single gold nanocrystal and θ and χ directional rotation mode. (**c**) Traces of diffraction spots from gold nanocrystals under saturated and supersaturated conditions. Blue and red traces indicate saturated and supersaturated solutions, respectively. Traces were subtracted by 184 and 267 traces in each condition. (**d,e**) Gold nanocrystal rotational angular displacement of θ (**d**) and χ (**e**) direction in saturated solutions. (**f,g**) Displacement of θ (**f**) and χ (**g**) direction in supersaturated solution.

**Figure 2:**
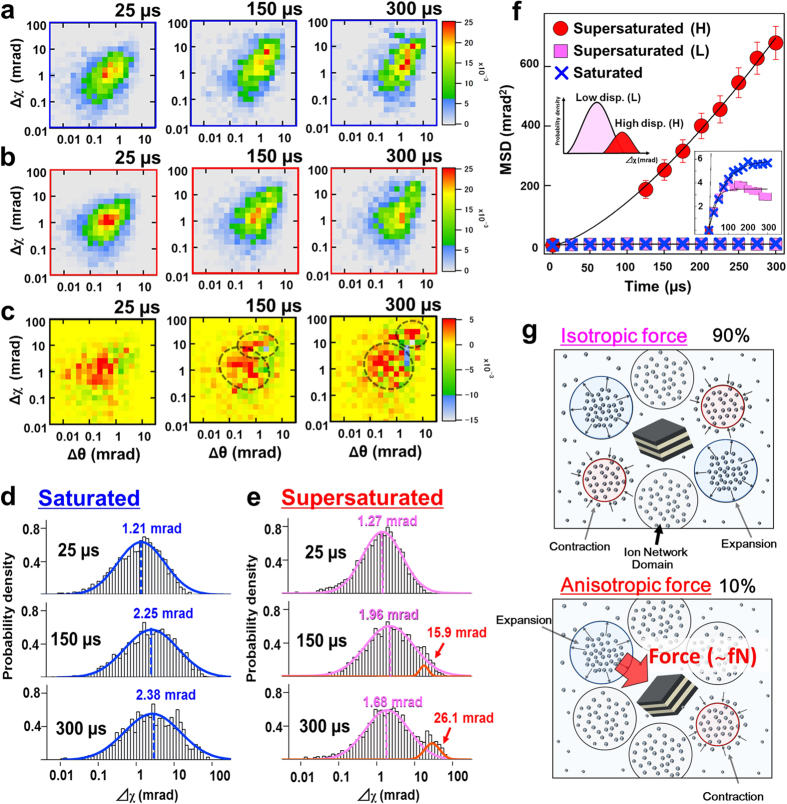
Statistical processing and regression analysis of DXT data. (**a–c**) Rotational motion map overlaid θ and χ angular displacement distribution of gold nanocrystals at 25 μs, 150 μs and 300 μs from saturated (**a**) and supersaturated (**b**) solutions. Differences in angular rotational motion maps that performed subtraction processes for the supersaturated from the saturated condition (**c**). (**d,e**) Normal distribution of displacement in χ direction during 25 μs, 150 μs and 300 μs. Blue and red figures show the saturated and supersaturated condition, respectively. (**f**) MSD curve from the peak position of the log-normal distribution. Red and pink circle plots indicate high and low displacement peak positions (H and L) in the supersaturated condition, respectively. Blue circle plots indicate the peak position in the saturated condition. (**g**) Schematic drawing of the estimated model of gold nanocrystals dispersed in the supersaturated solution. The upper figure shows the isotropic force, and the bottom figure indicates the anisotropic force by the expansion and contraction dynamics of INDs that affected a single gold nanocrystal.

**Figure 3:**
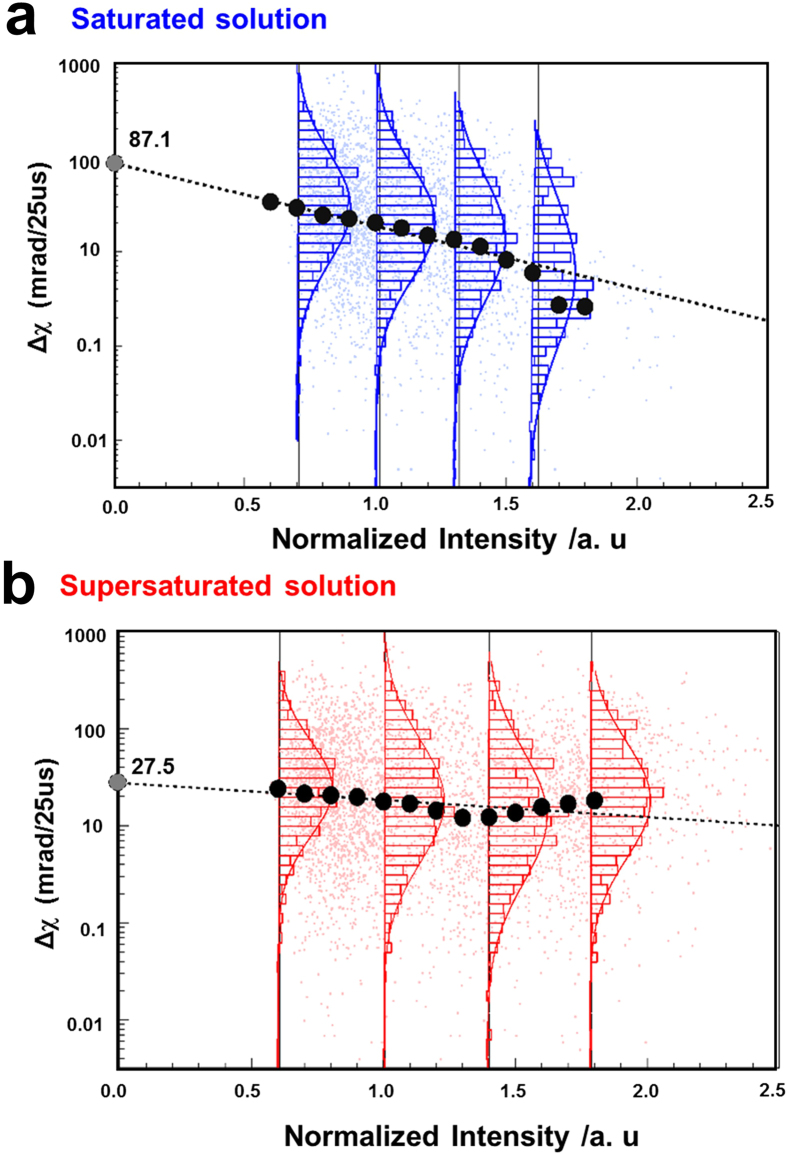
Size effect of gold nanocrystals in saturated and supersaturated solutions. (**a,b**) The relationship between gold nanocrystal size, as determined by the normalized intensity of each diffraction spot, and angular displacement in the χ direction at a 25 μs time interval.

**Figure 4:**
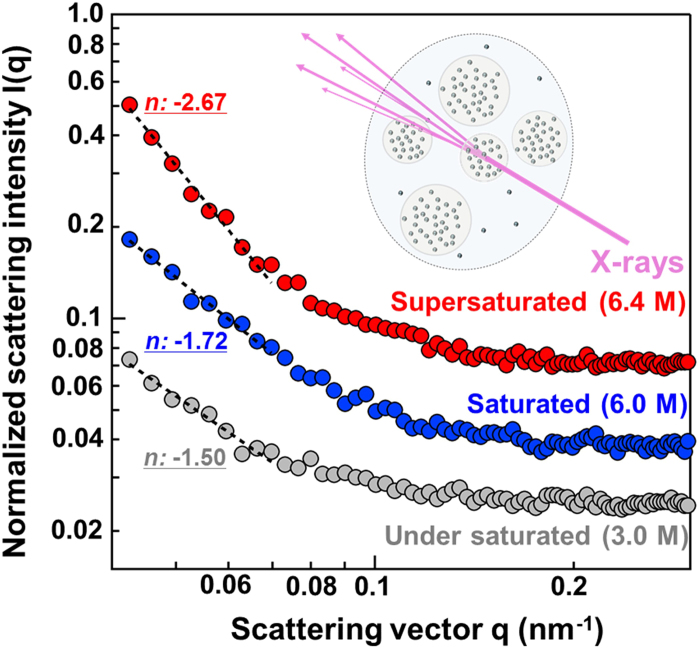
Large structure observations in supersaturated solutions by SAXS. (**a**) SAXS profile of normalized intensity I(q) and scattering vector q are shown on the vertical and horizontal axes, respectively. Red, blue and grey circle plots indicate 6.4, 6.0 and 3.0 M solutions of sodium acetate trihydrate. Inset values of *n* are the slopes of log-log linear fitting parameters ranging from 0.02 and 0.06 nm^−1^ of scattering vector q.
