# Supplementary material for: Time-resolved X-ray Tracking of Expansion and Compression Dynamics in Supersaturating Ion-Networks
Source: Sci Rep. 2015 Dec 14;5:17647. doi: 10.1038/srep17647 (PMC4677280; doi:10.1038/srep17647)
Supplement: Supplementary Figure S1 - S5 and, Table S1 - S2 [file srep17647-s1.pdf]

Time-resolved X-ray Tracking of Expansion and Compression Dynamics in Supersaturating Ion-Networks

Y. Matsushita[1], H. Sekiguchi[2] , K. Ichiyanagi[3], N. Ohta[2] K. Ikezaki[1] , Y. Goto[4], Y. C. Sasaki[1,2]

Corresponding author: Y. C. Sasaki
e-mail: ycsasaki@k.u-tokyo.ac.jp

1 Graduate School of Frontier Sciences, The University of Tokyo, 5-1-5 Kashiwanoha, Kashiwa City, Chiba, JAPAN

2 Japan Synchrotron Radiation Research Institute (JASRI) 1-1-1, Kouto, Sayo-cho, Sayo-gun, Hyogo 679-5198 JAPAN

3 High Energy Accelerator Research Organization, Photon Factory, 1-1 Oho, Tsukuba City, Ibaraki, JAPAN

4 Institute for Protein Research, Osaka University,3-2, Yamadaoka, Suita City, Osaka, JAPAN

Supplementary Information

-Supplementary Figure

-Supplementary Table

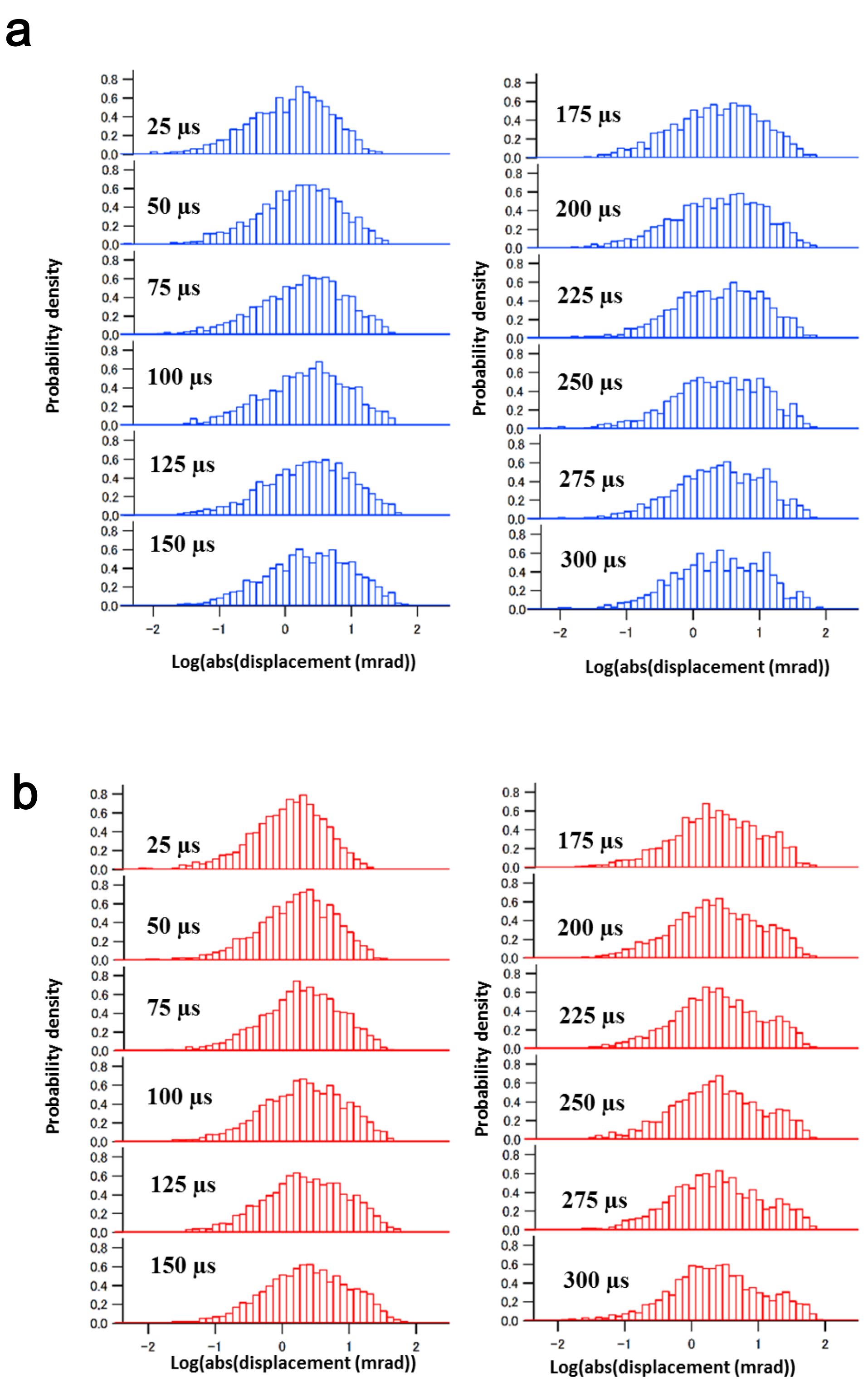

Supplementary Figure S1 | Distribution of angular displacement in χ direction at 25 µs interval until 300 µs. a, b, Angular displacement distribution of gold nanocrystals from saturated (**a**) and supersaturated condition (**b**) in units of 25 µs.

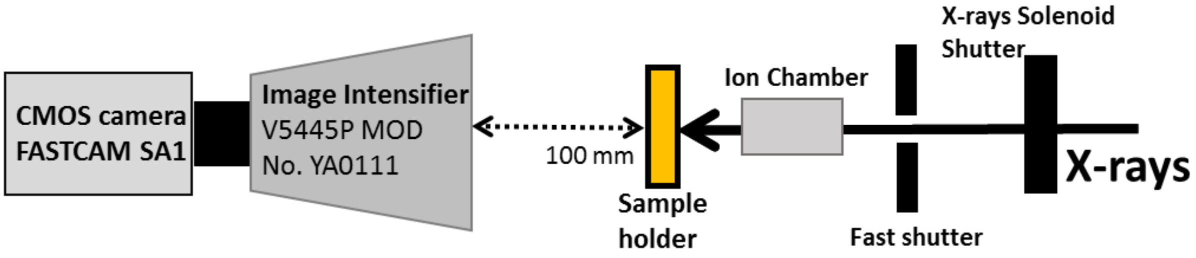

Supplementary Figure S2 | DXT measurement setup at SPring-8 BL40XU. X-ray from the BL40XU at SPring-8 with photon flux 10^{13} photons/sec and energy widths ranging from 14.0 – 16.45 keV (Undulator gap = 31.0 mm) were used for DXT measurement. The X-ray exposure time for the sample was adjusted 15 ms in order to avoiding radiation damage. Diffraction spots dynamics from the sample with 25 μs interval were detecting by CMOS camera (SA 1.1, Photron, Japan).

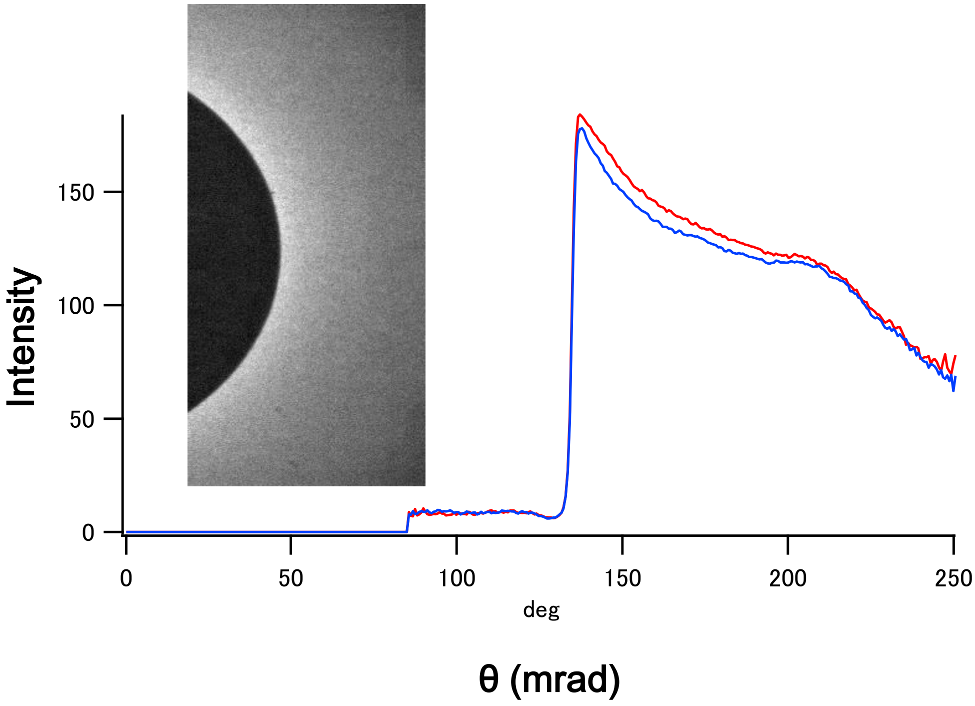

Supplementary Figure S3 | X-ray scattering intensity from supersaturated condition. X-ray scattering intensity from supersaturated conditions depend on the θ direction. This profile correspond to the back ground intensity of each diffraction spot intensity from DXT measurement.

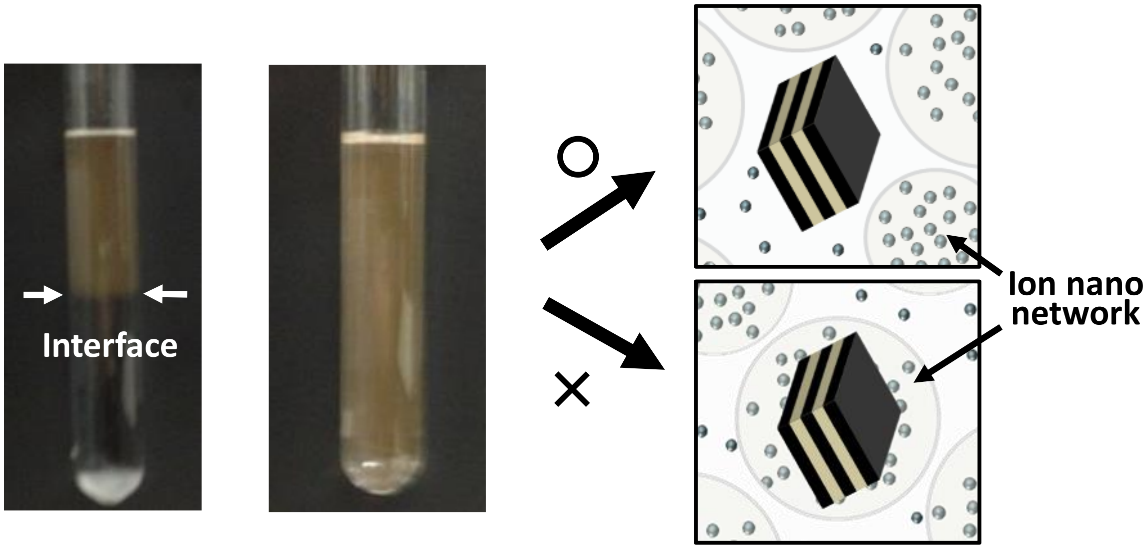

Supplementary Figure S4 | Macro dissolution and dispersion properties of gold nanocrystals in supersaturated solution. Gold nanocrystals dispersed in entire solution systems as shown as surface Plasmon resonance from gold nanocrystals were confirmed in throughout entire solution systems.

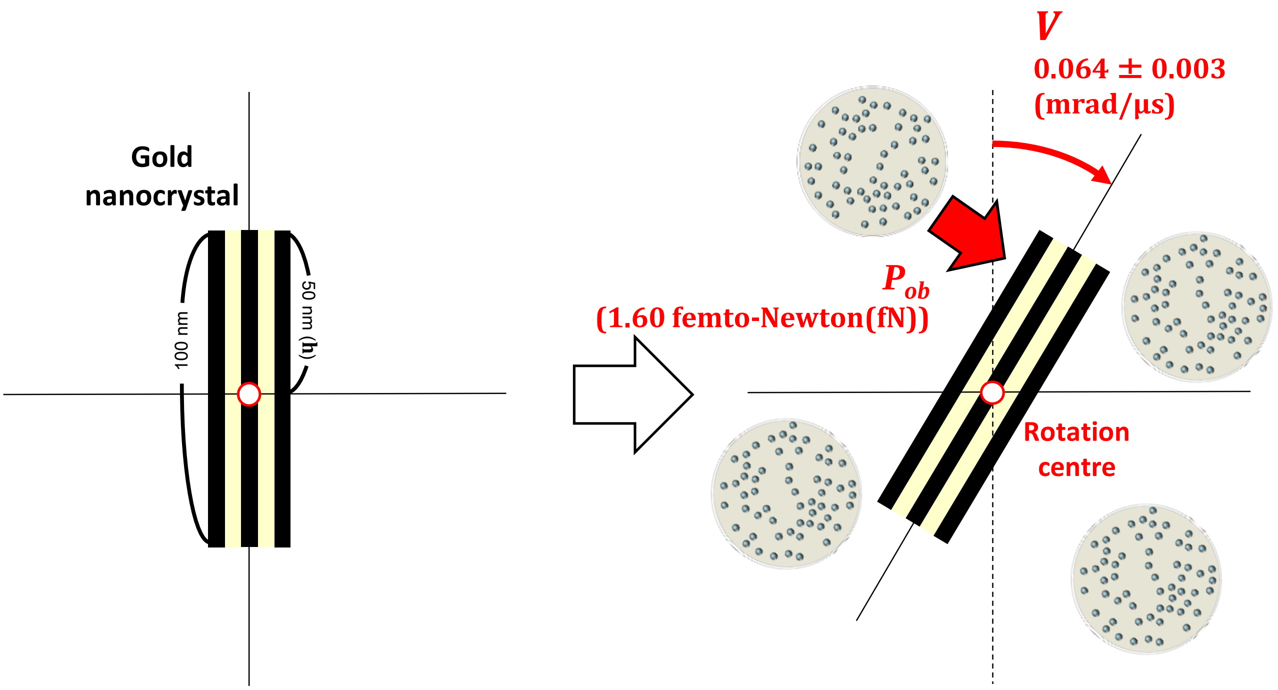

$$P_{ob} = \Gamma / h = 2\pi C_{rd} W / h$$

$$C_{rd} = \pi \eta h^3$$

$$W = V/2\pi$$

P_{ob}: Observed force pressure

h: Radius of a gold nanocrystal

Γ: Rotational Torque

η: Viscosity of sodium acetate

V: Rotational velocity

C_{rd} : Observed force pressure

Supplementary Figure S5 | The assumption of the gold nanocrystal dynamics to calculate the observerd force pressure P_{ob} . The calculation of the observed force pressure P_{ob} , we based on the assumption that the rotational centre is the centre of a gold nanocrystal. The parameters for the calculation were used by the referencse[21, 22].

	Chi Square χ^2		
	Saturated	Supersaturated	
	Single peak	Single peak	Double peaks
**25 μs**	0.132484	0.129273	-
**50 μs**	0.109049	0.188273	-
**75 μs**	0.164126	0.127208	-
**100 μs**	0.129986	0.179066	-
**125 μs**	0.0814483	0.122922	0.091808
**150 μs**	0.128428	0.222114	0.18167
**175 μs**	0.136766	0.266943	0.19588
**200 μs**	0.144686	0.225762	0.13692
**225 μs**	0.173474	0.262925	0.13337
**250 μs**	0.204597	0.281508	0.17583
**275 μs**	0.202261	0.260135	0.15406
**300 μs**	0.253876	0.277381	0.13811

Supplementary Table S1 | Chi square value χ^2 from regression analysis of log-normal distribution for saturated and supersaturated condition. Chi square value of saturated condition were obtained by single log-normal distribution fitting. Supersaturated condition were calculated by single and double log-normal distribution.

Fitting parameter of SAXS profile		
Sample concentration	n	A
6.4 M	-2.67 ± 0.12	-4.20 ± 0.15
6.0 M	-1.72 ± 0.09	-3.10 ± 0.11
3.0 M	-1.50 ± 0.10	-3.20 ± 0.13

Supplementary Table S2 | Fitting parameter from SAXS profile. Parameter from Log – Log linear fitting [$I(q) = nq + A$] process ranging from 0.04 – 0.06 nm^{-1} of scattering vector q. Where the value of n and A corresponded to the slope that related to structure size and intercept of SAXS profile respectively.

a

	V (mrad/μs)	D (mrad^2/μs)
**High displacement (H)**	0.064±0.003	0.28±0.03

b

	V (mrad/μs)	h (nm)	Γ (fN nm)	P_{ob} (fN)
**High displacement (H)**	0.064±0.003	50	76.56±3.59	1.60±0.07

Supplementary Table S3 | Parameters of anisotropic force by rotational velocity *V* from MSD curve of high displacement (H)

V is the rotational velocity from fitting process for high displacement mode (H) of gold nanocrystals. h is the gold nanocrystal radius. Γ is a torque from velocity V and estimated viscosity η of sodium acetate supersaturated solution (6.4 M). P_{ob} is a observed anisotropic force that affecting a single gold nanocrystals.
